# Supplementary material for: A Closer Look at Changes in High-Risk Food-Handling Behaviors and Perceptions of Primary Food Handlers at Home in South Korea across Time
Source: Foods. 2020 Oct 13;9(10):1457. doi: 10.3390/foods9101457 (PMC7602069; doi:10.3390/foods9101457)
Supplement: Supplementary file 1 [file foods-09-01457-s001.pdf]

foods

MDPI

Table S1. Detailed data regarding the discordance between consumers' food safety perceptions and behaviors (Risk perception-behavior gap).

Topic	Questions and Choices	Percent of Respondents or Answers		*p*-value [1]
		Year 2010 (N = 609)	Year 2019 (N = 605)	
Gap between the consumers' perceptions and behaviors regarding the storage of perishable foods without washing and trimming	Q1. Do you think storing raw materials including fruits, vegetables, meat, and fish/shellfish without preparation (e.g. washing or trimming) is hazardous?			
	Completely hazardous	27.8%	25.5%	0.366
	Mostly hazardous	44.0%	42.5%	0.591
	Moderate	21.3%	25.0%	0.136
	Mostly safe	5.9%	5.8%	0.925
	Completely safe	1.0%	1.3%	0.582
	<Analysis of the combined responses> [2]			
	Completely hazardous + Mostly hazardous	71.8%	67.9%	0.147
	Completely safe + Mostly safe	6.9%	7.1%	0.886
	Q7. Do you wash or trim meat, fish, fruits, and vegetables before storage?			
	Always	22.5%	7.9%	0.000
	Frequently	29.2%	27.8%	0.573
	Sometimes	15.8%	30.6%	0.000
	Seldom	20.7%	25.0%	0.076
	Never	11.8%	8.8%	0.079
	<Analysis of the combined responses> [3]			
	Always + Frequently	51.7%	35.7%	0.000
	Never + Seldom	32.5%	33.7%	0.655
Gap between the consumers' perceptions and behaviors regarding the frozen foods	Q2. Do you think thawing frozen foods at room temperature is hazardous?			
	Completely hazardous	26.4%	17.4%	0.000
	Mostly hazardous	36.8%	30.7%	0.026
	Moderate	22.0%	35.0%	0.000
	Mostly safe	12.0%	14.7%	0.163
	Completely safe	2.6%	2.1%	0.585
	Do not know/no response	0.2%	0.0%	0.319
	<Analysis of the combined responses> [2]			
	Completely hazardous + Mostly hazardous	63.2%	48.1%	0.000
	Completely safe + Mostly safe	14.6%	16.9%	0.283
	Q12. How do you defrost frozen foods?			
	Do not thaw	4.4%	3.0%	0.179
	Place in the refrigerator for 1–2 days before it is cooked	17.1%	23.0%	0.010
	Use microwave oven	25.0%	15.5%	0.000
	Dip in water at room temperature	16.6%	14.9%	0.414
	Place on counter at room temperature	36.9%	43.6%	0.018

Gap between the consumers' perceptions and behaviors regarding the leftovers	Q5. Do you think storing leftovers at room temperature is hazardous?			
	Completely hazardous	20.7%	21.5%	0.733
	Mostly hazardous	43.3%	39.3%	0.156
	Moderate	23.3%	32.4%	0.000
	Mostly safe	11.2%	6.3%	0.003
	Completely safe	1.5%	0.5%	0.084
	<Analysis of the combined responses> [2]			
	Completely hazardous + Mostly hazardous	64.0%	60.8%	0.248
	Completely safe + Mostly safe	12.6%	6.8%	0.001
	Q18. How do you store hot and leftover foods?			
	Do not know/no response	0.3%	0.0%	0.158
	Store immediately in the refrigerator	1.6%	3.5%	0.043
	Store in refrigerator after chilling in cold water	2.5%	3.5%	0.301
	Store at room temperature	39.6%	24.1%	0.000
	Store in refrigerator after chilling at room temperature	56.0%	68.9%	0.000

[1] *p*-value was provided according to the results of the Kruskal–Wallis test conducted for the analysis on the differences of responses in each answer option between Year 2010 and Year 2019. [2] To identify the difference between the perception of hazards (i.e., Completely hazardous + Mostly hazardous) and the perception of safety (i.e., Completely safe + Mostly safe), answer options were combined. [3] To identify the difference between positive responses (i.e., Always + Frequently) and negative responses (i.e., Never + Seldom), answer options were combined.

Table S2. Detailed data regarding the proper risk perception (i.e., the correct response in line with conventional food safety guidelines) and behaviors of consumers.

Topic	Questions and Choices	Percent of Respondents or Answers		*p*-value [1]
		Year 2010 (N = 609)	Year 2019 (N = 605)	
Proper behaviors of consumers in accordance with their perceptions regarding the washing hands	Q3. Do you think to touch cooked foods after handling raw materials without washing your hands is hazardous?			
	Completely hazardous	30.2%	28.9%	0.623
	Mostly hazardous	53.2%	48.9%	0.136
	Moderate	13.3%	17.4%	0.050
	Mostly safe	3.0%	3.3%	0.726
	Completely safe	0.3%	1.5%	0.033
	<Analysis of the combined responses> [2]			
	Completely hazardous + Mostly hazardous	83.4%	77.9%	0.014
	Completely safe + Mostly safe	3.3%	4.8%	0.182
	Q11. Do you wash your hands to handle other foods after handling meat, fish/shellfish, or eggs?			
	Always	44.7%	41.3%	0.240
	Frequently	29.6%	25.8%	0.142
	Sometimes	8.7%	17.5%	0.000
	Seldom	12.2%	10.4%	0.339
	Never	4.8%	5.0%	0.873
	Do not know/no response	0.2%	0.0%	0.319
	<Analysis of the combined responses> [3]			
	Always + Frequently	74.2%	67.1%	0.007
	Never + Seldom	16.9%	15.4%	0.466
Proper behaviors of consumers in accordance with their perceptions regarding the incomplete cooking	Q4. Do you think to incompletely cook for meat, fish/shellfish, and eggs is hazardous?			
	Completely hazardous	46.0%	38.3%	0.007
	Mostly hazardous	44.0%	41.7%	0.408
	Moderate	9.0%	16.4%	0.000
	Mostly safe	0.8%	2.5%	0.023
	Completely safe	0.0%	1.2%	0.008
	Do not know/no response	0.2%	0.0%	0.319
	<Analysis of the combined responses> [2]			
	Completely hazardous + Mostly hazardous	90.0%	80.0%	0.000
	Completely safe + Mostly safe	0.8%	3.6%	0.001
	Q16. Do you fully cook foods like meat, chicken, and fish/shellfish for eating?			
	Always	56.8%	47.6%	0.001
	Frequently	37.3%	38.0%	0.790
	Sometimes	3.4%	11.7%	0.000
	Seldom	1.8%	2.3%	0.534
	Never	0.3%	0.3%	0.995
	Do not know/no response	0.3%	0.0%	0.158
	<Analysis on the combined responses> [3]			

	Always + Frequently	2.1%	2.6%	0.561
	Never + Seldom	94.1%	85.6%	0.000
Proper behaviors of consumers in accordance with their perceptions regarding reheating leftovers	Q6. Do you think to eat leftover without re-heat is hazardous?			
	Completely hazardous	25.9%	23.3%	0.286
	Mostly hazardous	50.9%	43.5%	0.010
	Moderate	17.4%	26.6%	0.000
	Mostly safe	5.3%	6.1%	0.517
	Completely safe	0.3%	0.5%	0.649
	Do not know/no response	0.2%	0.0%	0.319
	<Analysis of the combined responses> [2]			
	Completely hazardous + Mostly hazardous	76.8%	66.8%	0.000
	Completely safe + Mostly safe	5.6%	6.6%	0.454
	Q19. Do you re–heat leftovers for eating?			
	Always	46.3%	50.6%	0.136
	Frequently	45.6%	34.4%	0.000
	Sometimes	6.7%	12.2%	0.001
	Seldom	0.5%	1.5%	0.080
	Never	0.5%	1.3%	0.127
	Do not know/no response	0.3%	0.0%	0.158
	<Analysis of the combined responses> [3]			
	Always + Frequently	92.0%	85.0%	0.000
	Never + Seldom	1.0%	2.8%	0.020

[1] *p*-value was provided according to the results of the Kruskal–Wallis test conducted for the analysis on the differences of responses in each answer option between Year 2010 and Year 2019. [2] To identify the difference between the perception of hazards (i.e., Completely hazardous + Mostly hazardous) and the perception of safety (i.e. Completely safe + Mostly safe), answer options were combined. [3] To identify the difference between positive responses (i.e., Always + Frequently) and negative responses (i.e. Never + Seldom), answer options were combined.
